# Peer Review of “Machine Learning and Medication Adherence: Scoping Review”

**DOI:** 10.2196/33963

**Published:** 2021-11-24

**Authors:** Yu Heng Kwan

**Affiliations:** 1 Duke-NUS Medical School Singapore Singapore

*This is a peer-review report submitted for the paper “Machine Learning and Medication Adherence: Scoping Review”.*

## Round 1 Review

### General Comments

This paper [1] aims to categorize and summarize literature focused on using machine learning for medication compliance activities. There are major concerns associated with this paper.

### Specific Comments

#### Major Comments

1. The aim does not feel like an actual aim. I would suggest saying things like “aim to do a scoping review on... and categorize and summarize...”
2. You should state the design in the methods. In addition, you should state clearly the inclusion and exclusion criteria. As of now, the inclusion and exclusion criteria are too broad to do a robust review.
3. Is it adherence or compliance? The frequent change of terms makes it hard to understand what the authors want to do. They are very different fundamentally.
4. Limitations should be before the Conclusion.
5. The paper lacks Figure 1: the number of articles screened/reviewed.
6. Figure 2 is not right; there are many overlapping diseases in each category.
7. Short forms are not well explained or mentioned in the tables.

Thank you for allowing me to review the paper.

## Round 2 Review

I am appreciative that the authors are willing to do the changes, and the manuscript has improved vastly.

Although this is a scoping review, I would like to find out how the author ensured robustness and reproducibility. As of now, with the study design largely using one author, there is no way to assess if the paper selection is robust or independent. I strongly feel that there is a need for 2 authors to independently select studies, even though it is a scoping review, to give this review some robustness. If not, how different will the results be compared to a narrative review?

I hope to see how the author justifies this step here.

## Round 3 Review

Thanks for revising. Appreciate the effort.

May I suggest shortening the Discussion further? The limitations should be in the second to last paragraph. The last paragraph should be the conclusion of this study. It will be great if the Discussion is shortened to 4-5 paragraphs max.
